# Left ventricular high frame rate echo-particle image velocimetry: clinical application and comparison with conventional imaging

**DOI:** 10.1186/s12947-022-00283-4

**Published:** 2022-04-26

**Authors:** Mihai Strachinaru, Jason Voorneveld, Lana B. H. Keijzer, Daniel J. Bowen, Ferit O. Mutluer, Folkert ten Cate, Nico de Jong, Hendrik J. Vos, Johan G. Bosch, Annemien E. van den Bosch

**Affiliations:** 1 Department of Biomedical Engineering, Erasmus MC, Rotterdam, Netherlands; 2 Department of Cardiology, Erasmus MC, Rotterdam, Netherlands; 3 Department of Medical Physics, Spaarne Gasthuis, Haarlem, Netherlands; 4 Amsterdam UMC, Department of Radiology and Nuclear Medicine, Amsterdam, Netherlands; 5 Present Address: Yeditepe University Hospital, Department of Cardiology, Istanbul, Turkey

**Keywords:** High frame rate contrast echocardiography, Echo-particle image velocimetry

## Abstract

**Background:**

*Echo-Particle Image Velocimetry* (echoPIV) tracks speckle patterns from ultrasound contrast agent(UCA), being less angle-sensitive than colour Doppler. High frame rate (HFR) echoPIV enables tracking of high velocity flow in the left ventricle (LV). We aimed to demonstrate the potential clinical use of HFR echoPIV and investigate the feasibility and accuracy in patients.

**Methods:**

Nineteen patients admitted for heart failure were included. HFR contrast images were acquired from an apical long axis view (ALAX), using a fully-programmable ultrasound system. A clinical UCA was continuously infused with a dedicated pump. Additionally, echocardiographic images were obtained using a clinical system, including LV contrast-enhanced images and pulsed-wave (PW) Doppler of the LV inflow and outflow in ALAX. 11 patients underwent CMR and 4 cardiac CT as clinically indicated. These CMR and CT images were used as reference. In 10 patients with good echoPIV tracking and reference imaging, the intracavitary flow was compared between echoPIV, conventional and UCA echocardiography.

**Results:**

EchoPIV tracking quality was good in 12/19 (63%), moderate in 2/19 (10%) and poor in 5/19 (26%) subjects. EchoPIV could determine inflow velocity in 17/19 (89%), and outflow in 14/19 (74%) patients. The correlation of echoPIV and PW Doppler was good for the inflow (R^2^ = 0.77 to PW peak; R^2^ = 0.80 PW mean velocity) and moderate for the outflow (R^2^ = 0.54 to PW peak; R^2^ = 0.44 to PW mean velocity), with a tendency for echoPIV to underestimate PW velocities. In selected patients, echoPIV was able in a single acquisition to demonstrate flow patterns which required multiple interrogations with classical echocardiography. Those flow patterns could also be linked to anatomical abnormalities as seen in CMR or CT.

**Conclusion:**

HFR echoPIV tracks multidirectional and complex flow patterns which are unapparent with conventional echocardiography, while having comparable feasibility. EchoPIV tends to underestimate flow velocities as compared to PW Doppler. It has the potential to provide in one acquisition all the functional information obtained by conventional imaging, overcoming the angle dependency of Doppler and low frame rate of classical contrast imaging.

**Supplementary Information:**

The online version contains supplementary material available at 10.1186/s12947-022-00283-4.

## Introduction

Blood flow in the heart is classically imaged by using colour Doppler, which has some inherent disadvantages: angle-dependency, relatively low frame rate, low velocity range and semi-quantitative nature. Recent advances in ultrasound technology may offer new opportunities for imaging the dynamics of the blood flow in the heart and vessels.

New ultrasound techniques, generally referred to as vector flow imaging, can estimate the location, direction and magnitude of velocity vectors describing the flow in a specific region of interest. One such technique, *Echo-Particle Image Velocimetry* (echoPIV), tracks the speckle of ultrasound contrast agent (UCA) microbubbles [1–3]. One of the limitations of conventional echoPIV is the inability to accurately resolve high velocities, because of the relatively low frame rates permitted by conventional line-scanning based ultrasound imaging (maximum ~ 100 Hz).

Current clinical ultrasound systems use a line-by-line approach in order to construct an echocardiographic image, and lines are generated through focused beams. In order to increase the frame rate and maintain a low acoustic pressure we used a diverging-wave acquisition scheme [4–8]. While in non-enhanced high frame rate (HFR) echocardiography, the use of nonfocused beams generates little harmonic signal from the tissue, by adding UCA the harmonic signal comes from the interaction of the nonfocused beams with the UCA microbubbles [5]. This means that by using a pulse inversion scheme we can very effectively suppress the tissue signal, and retain the harmonic signal generated by the UCA [5, 6].

High frame rate echoPIV (HFR echoPIV), using diverging-wave transmit sequences, allows for frame rates in the kHz range and makes tracking of multi-directional fast flow in the left ventricle (LV) possible [4–7].

In this study we demonstrate the potential clinical application of the new HFR approach to echoPIV, and test its feasibility and accuracy in patients with a broad range of pathologies.

## Methods

Nineteen consecutive subjects were prospectively included, selected from patients admitted for heart failure symptoms, regardless of the underlying cause. The purpose of this selection was to test the method in a real-life setting, with everyday regular patients.

**Fig. 1:**
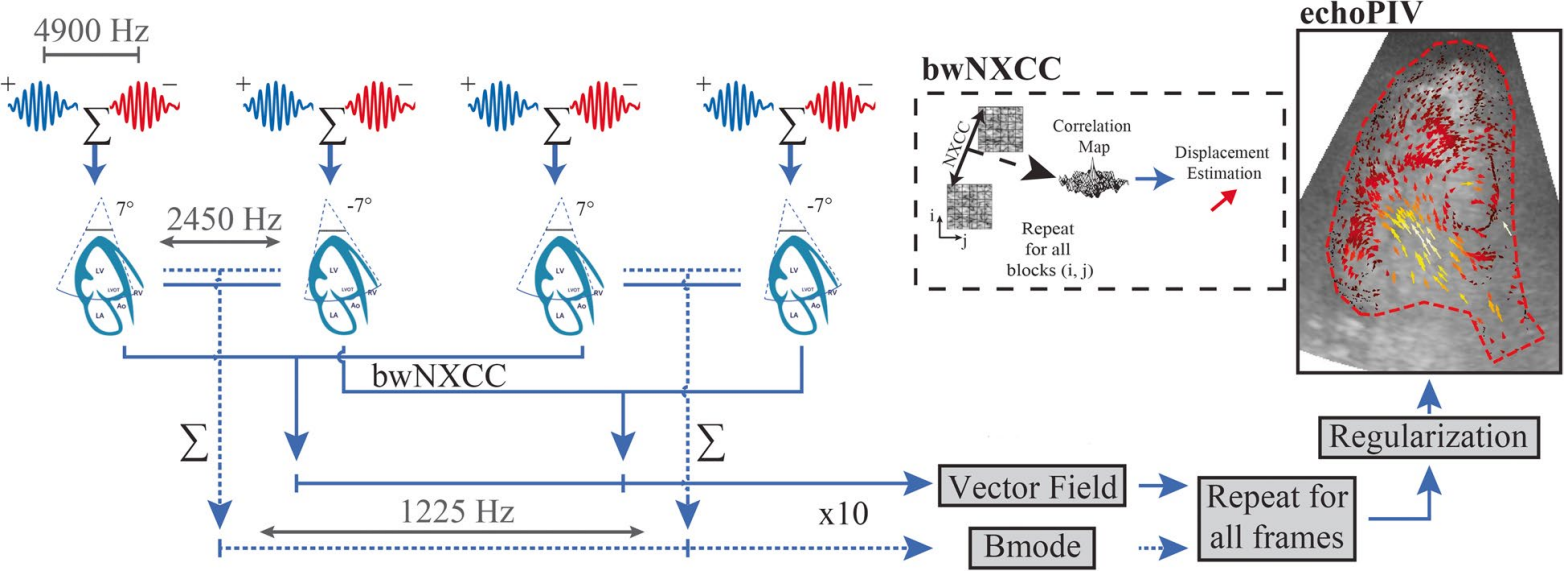
Flow diagram of echoPIV processing. A two-angled diverging-wave pulse-inversion sequence is used at a pulse repetition frequency of 4900 Hz. Adjacent phase-inverted pulses are summed to suppress tissue clutter. The two angles are coherently summed to produce a BMode image; while block-wise normalized cross-correlation (bwNXCC) is performed between like angles to obtain the velocity vectors. After averaging 10 frames a single echoPIV frame is produced. Once all frames have been processed, the velocity field is regularized with spatial and temporal smoothing. Borders of the LV endocardium were manually drawn to fit the end diastolic shape (echoPIV image, red-dashed lines, Additional file 1). (Additional file 1 = flow in a normal heart)

HFR contrast images were acquired, in apical long axis view (ALAX, ensuring simultaneous visualization of LV inflow and outflow), using a fully programmable ultrasound system (Verasonics Vantage 256, with a P4-1 probe). We used four repeated diverging-waves, with two alternating polarity transmits (+ 7˚, -7˚), at a depth of 12 cm (Fig. 1, Additional file 1), providing an effective frame rate of 1225 fps [6–8].

**Fig. 2:**
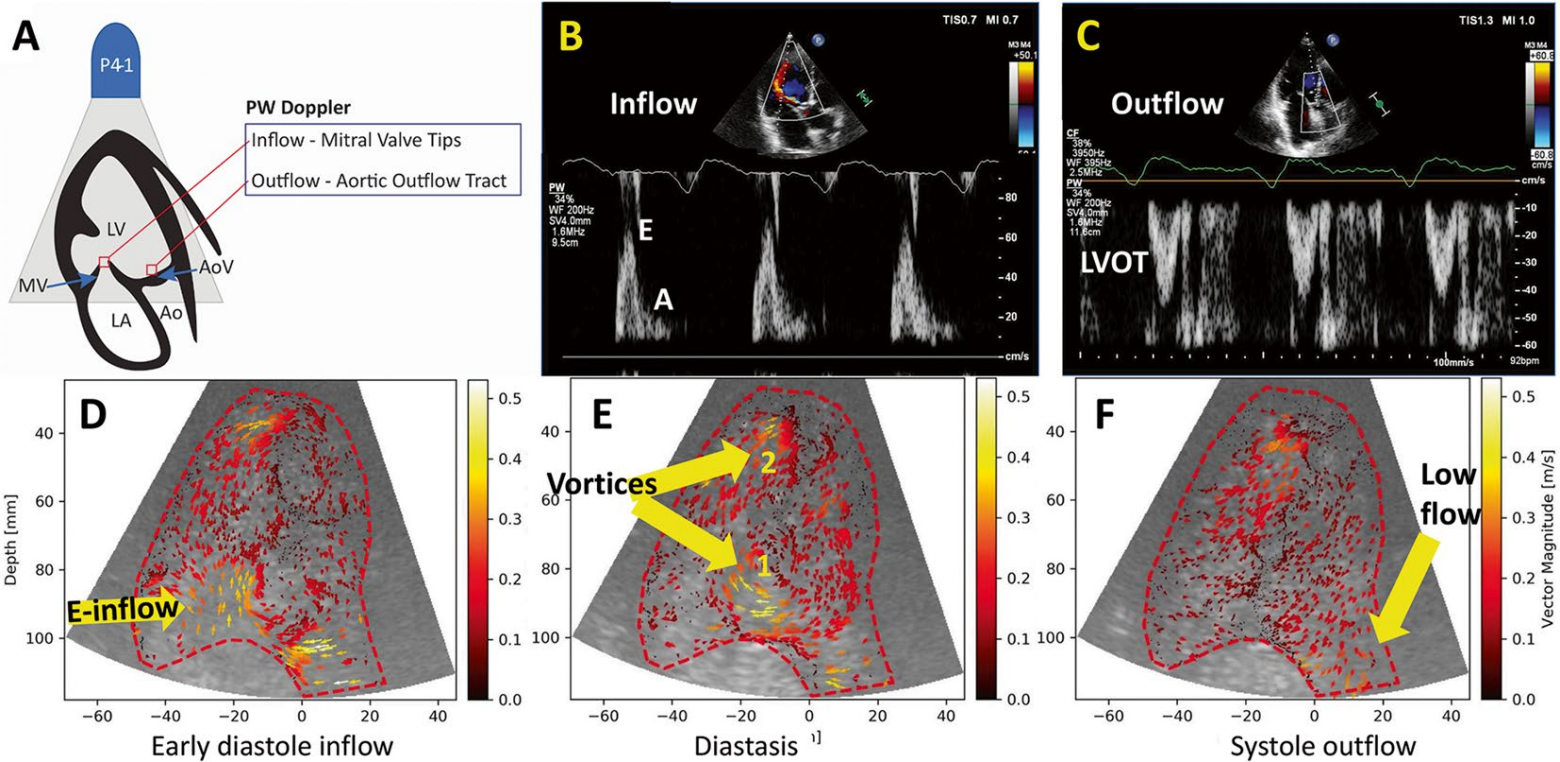
Image acquisition. A: apical long axis view with the position of the Doppler and echoPIV interrogation points; B: PW Doppler of the inflow in a heart failure patient. Early diastolic flow (E wave) was measured in this study, the post-atrial contraction flow (A wave) was not considered; C: left ventricle outflow tract (LVOT) PW Doppler flow in the same heart failure patient, demonstrating low output (low velocity); D: echoPIV tracking of the early diastolic inflow in the same patient; E: mid-diastolic vortex formation in the LV. Two main diastolic vortices (1,2) can be seen; F: low velocity outflow, confirmed by the PW Doppler (C)

UCA was infused using a Bracco VueJect pump (VueJect BR-INF 100, Bracco Imaging, Milan Italy), aiming for a steady-state relatively low-concentration of bubbles into the LV, allowing for the tracking of individual bubble motion between frames [8]. In the same session, complete echocardiographic studies were obtained using a clinical ultrasound system (Philips Epiq 7, with a X5-1 probe), including LV UCA. Non-contrast pulsed-wave (PW) Doppler were also obtained in ALAX (Fig. 2) from the mitral valve tips (inflow) and the LV outflow tract (outflow). This view was chosen because its ability to visualize both the inflow and outflow of the LV in a single echocardiographic plane, and has furthermore been widely used by previous vector flow imaging investigations.

### EchoPIV processing and analysis

The beamforming and detection of the vector field have already been described in previous works [6–8]. The HFR radiofrequency data was converted offline to B-mode images by successive filtering and beamforming onto a polar coordinate system, using the Verasonics beamformer. The resulting polar beamformed IQ data were used for echoPIV detection. A static boundary mask was manually drawn on the end-diastolic endocardial contour of the LV. EchoPIV vector detection was performed inside this boundary, in order to limit the noise and tissue signal, and reduce the processing time. The echoPIV velocity estimation after envelope detection was performed using a PIV algorithm developed in MATLAB (R2019a, MathWorks, Natick, MA, USA). This algorithm divides the image into equally sized overlapping blocks; then, normalized cross correlation (NXCC, Fig. 1) was performed per block between subsequent frames, to obtain the local displacement between frames. The algorithm performs four iterations of the NXCC block displacement analysis, increasing the resolution and decreasing the estimation error. The resulting velocity data were scan converted for visualization inside the boundary mask, by using the vector projectile imaging technique [9].

### Other conventional imaging

All patients underwent conventional echocardiographic contrast imaging, using the same clinical ultrasound system, during the same imaging session (HFR and clinical imaging). Complementary imaging studies: (cardiac magnetic resonance (CMR) and cardiac computed tomography (CCT)) were performed during the hospital stay, if clinically indicated.

In cases where reference images (CT or CMR) were available, the intracavitary flow direction and particular vortices or jets as seen with HFR echoPIV could be visually related to confirmed anatomical abnormalities.

HFR image quality and echoPIV tracking were qualitatively assessed offline by two independent observers (JV- engineer specialized in clinical ultrasound and FM – clinical cardiologist) and graded as good, moderate and poor. In case of disagreement, cases were reviewed by an experienced imaging cardiologist (MS). The highest velocity of the inflow and outflow were determined by the automated tracking algorithm of the HFR echoPIV, and also measured by the peak and mean modal velocity of the conventional PW Doppler. For the purpose of this comparison, the only diastolic inflow velocity we measured was the early diastolic (E wave), which is present in all patients regardless of the underlying rhythm.

### Statistics

Data normality was assessed using a Shapiro–Wilk test. Continuous variables were represented by mean ± standard deviation (SD) or as median (interquartile range = IQR), and discrete variables as proportions (%). Differences between percentages were assessed by using the Chi square test. Correlations were investigated using linear regression. The bias and limits of agreement between methods were computed using Bland–Altman plots. Statistical significance was considered for *p* values < 0.05.

## Results

As the patients were selected in the order of their admission to the hospital for symptoms of heart failure, they exhibited a large palette of pathologies. Their baseline characteristics are presented in Table 1.

**Table 1:** baseline data and pathologies in the study group

Pathology	Number	CCT	CMR	Age [Years]	Gender [M/F]	BMI [kg/m^2^]	Heart rate [beats/min]	Systolic blood pressure [mmHg]	Diastolic blood pressure [mmHg]	EF [%]
Dilated cardiomyopathy										
Ischemic	5	1	1	66 (19–48)	4/1	25 ± 4	75 ± 6	101 ± 15	68 ± 10	25 (19–48)
Non-ischemic	6	1	4	63 (39–73)	2/4	23 ± 3	91 ± 14	92 ± 4	59 ± 5	43 (24–50)
Hypertrophic Cardiomyopathy	2	1	1	57 ± 8	1/1	32 ± 3	78 ± 1	126 ± 42	71 ± 16	50 ± 14
Restrictive Cardiomyopathy	2	0	2	67 ± 4	1/1	25 ± 7	68 ± 23	93 ± 11	58 ± 1	53 ± 11
Other										
Normal EF	1	1	0	60	1/0	29	67	128	71	60
Arrhythmia	2	0	2	40 ± 31	2/0	26 ± 4	87 ± 23	102 ± 8	74 ± 15	43 ± 4
Constrictive pericarditis	1	0	1	65	1/0	22	95	104	67	70

*Data is represented as average (*± *standard deviation) if normally distributed and as median (interquartile range) if abnormally distributed. BMI Body mass index, EF Ejection fraction, CMR Cardiac magnetic resonance, CCT: Cardiac computed tomography*

### Image quality and flow tracking

In the 19 patients, the image quality was good in 10 (53%), moderate in 5 (26%) and poor in 4 subjects (21%). The quality of the echoPIV tracking was good in 12 (63%), moderate in 2 (10%) and poor in 5 (26%).

Four patients underwent cardiac CT, 11 patients CMR, while 4 had no reference imaging test. In the 12 patients where the echoPIV tracking was good, the direction and the velocity of the intracavitary vortices during the cardiac cycle could be optimally visualized (Fig. 2, Additional file 2). From these 12 patients: 8 had CMR, 2 cardiac CT and 2 no reference imaging.

### Accuracy

The inflow velocity could be determined by echoPIV in 17/19 subjects (89%), and outflow in 14/19 (74%). EchoPIV tended to underestimate the maximal velocity as determined by PW.

**Fig. 3:**
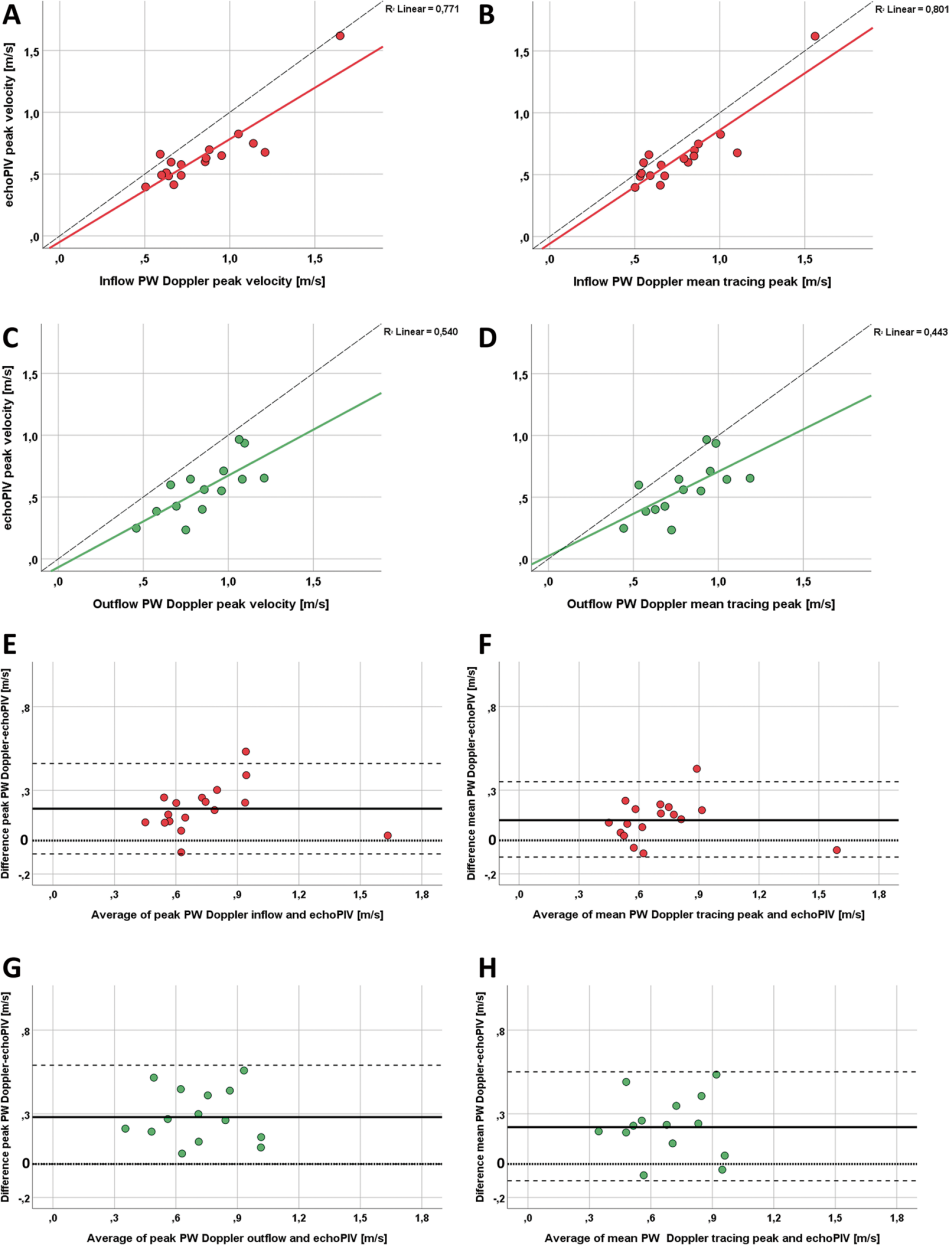
Statistical results. A-B: Linear correlation between echoPIV velocities of the LV inflow and peak (**A**) or mean (**B**) PW Doppler determined velocities; C-D: Linear correlation between echoPIV velocities of the LV outflow and peak (**A**) or mean (**B**) PW Doppler determined velocities. The correlation slope was computed (continuous line), and compared with the ideal correlation mid-line (dashed line). E–H: Bland–Altman plots showing the bias (horizontal continuous line) and limits of agreement (horizontal dashed lines) for the same comparisons as in the A-D plots

Doppler (Table 2, Fig. 3). The correlation of the two methods was good for the inflow (R^2^ = 0.77 with PW Doppler peak velocity tracing, *p* < 0.001 and R^2^ = 0.80 with PW mean velocity tracing, *p* < 0.001) and moderate for the outflow (R^2^ = 0.54, *p* < 0.001 for peak PW velocity and respectively 0.44, *p* = 0.009 for mean PW velocity). The lowest bias was also noted for the inflow as compared to the mean PW velocity tracing (Table 2).

**Table 2:** linear correlations and differences according to Bland–Altman analysis between echoPIV and peak PW Doppler velocities, and respectively peak of the mean tracing of PW Doppler velocities

*echoPIV versus PW Doppler maximal velocity*	**Linear correlation**				**Bland-Altmann**	
	*EchoPIV*	*PW Doppler*	*R*^*2*^	*P for correlation*	*Bias*	*LOA*
**Inflow [m/s]**	0.70 ± 0.27	0.84 ± 0.29	0.77	< 0.001	0.19 (27%)	-0.08 to 0.46
**Outflow [m/s]**	0.61 ± 0.22	0.85 ± 0.21	0.54	0.003	0.28 (45%)	-0.03 to 0.59
*echoPIV versus PW Doppler mean velocity*	*EchoPIV*	*PW Doppler*	*R*^*2*^	*P for correlation*	*Bias*	*LOA*
**Inflow [m/s]**	0.7 ± 0.27	0.77 ± 0.26	0.80	< 0.001	0.12 (17%)	-0.11 to 0.35
**Outflow [m/s]**	0.61 ± 0.22	0.79 ± 0.21	0.44	0.009	0.22 (36%)	-0.11 to 0.55

HFR echoPIV also detected intracavitary jets that were not directly visible on the corresponding apical long axis colour Doppler classical echocardiography. Furthermore, those functional phenomena could be related to anatomical abnormalities seen in the associated CCT/CMR images

We shortly present hereafter four clinical examples where the added value of the HFR-echoPIV is illustrated and the findings are confirmed by other imaging modalities.

## Patient 1: Left ventricle outflow tract (LVOT) narrowing not visible on 2D echocardiography.

**Fig. 4:**
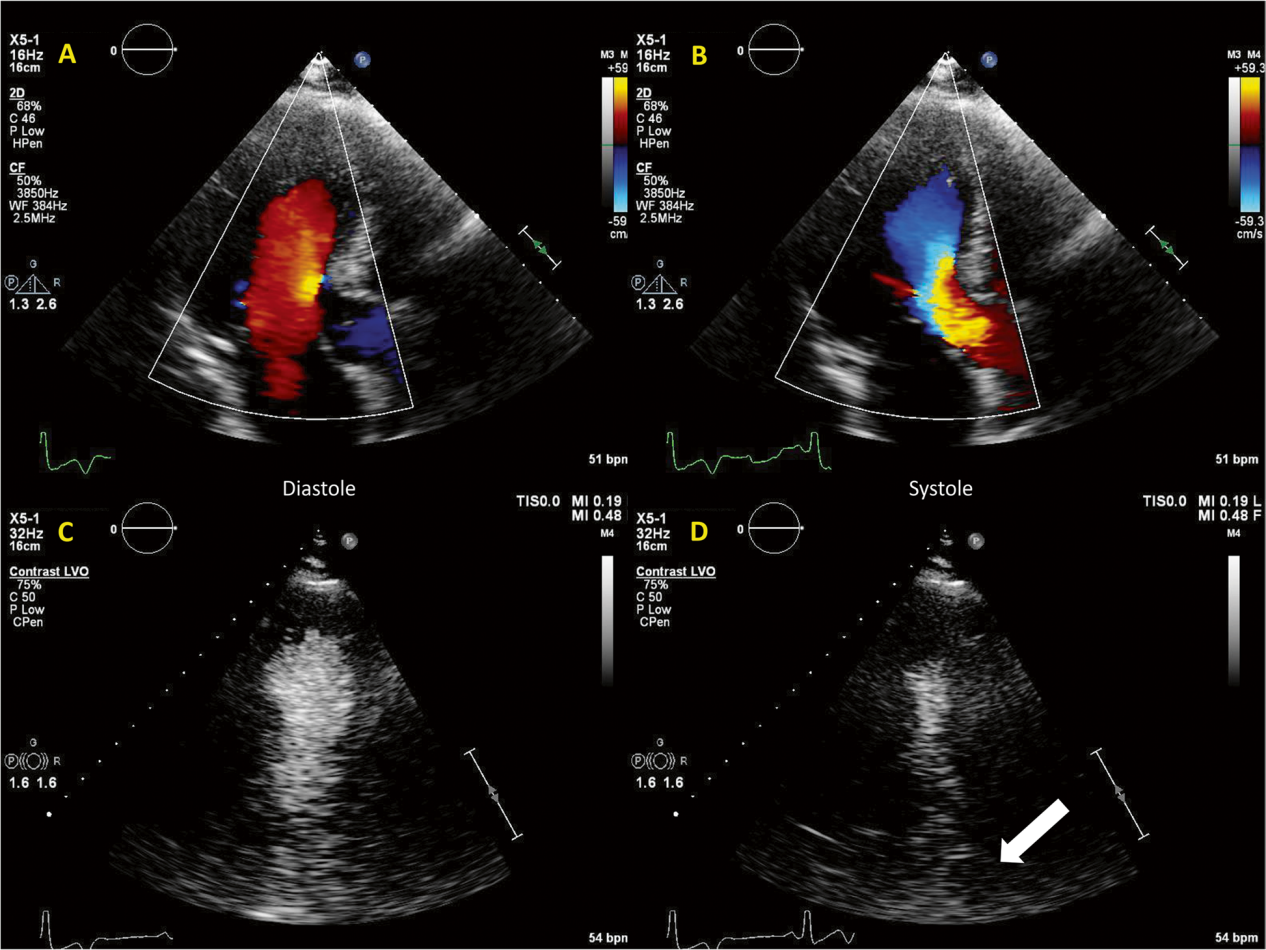
**Patient 1.** A, B: color Doppler (Additional file 2). There is no visible abnormal flow acceleration in the LVOT; C, D: Classical LV contrast imaging (Additional file 3) suggested cavity collapse towards LVOT (arrow)

**Fig. 5:**
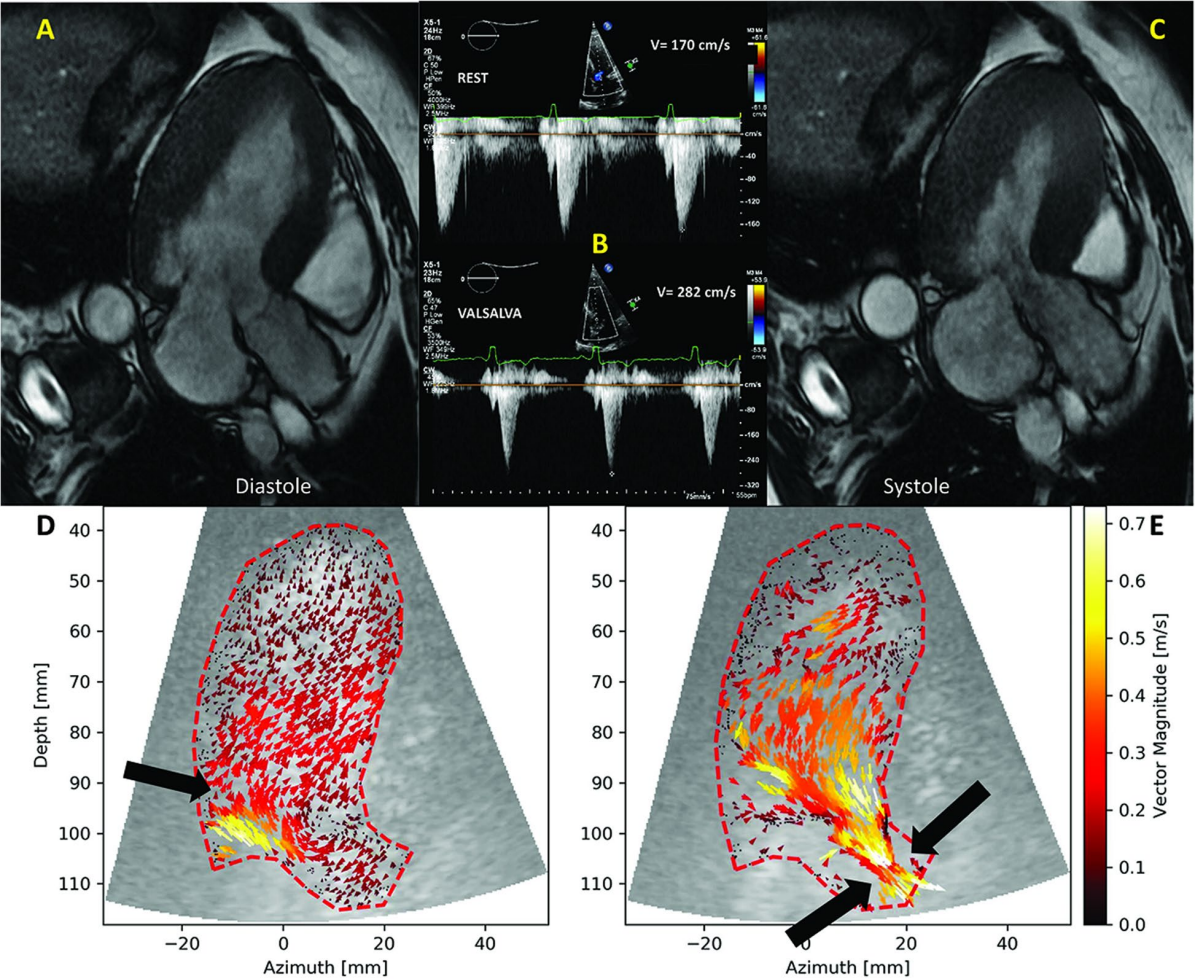
**Patient 1.** A, C: CMR in diastole and systole, highly suggestive for a hypertrophic cardiomyopathy; flow acceleration in the LVOT with a tendency to luminal obliteration in mid-systole; B: CW Doppler of the LVOT at rest (upper) and during Valsalva manoeuver (lower).The high-velocity flow could also be detected by careful interrogation with continuous-wave Doppler. D: HFR echoPIV in early diastole. Early diastolic inflow (black arrow) had higher velocity and an vertical direction (Additional file 5), and echoPIV revealed the lacking of the mid-LV vortex that normally appears in healthy individuals (Additional file 1), probably as a result of diastolic dysfunction.; E: HFR echoPIV in mid-systole. LVOT flow acceleration and narrowing (black arrows)

A 52-year-old female patient with hypertrophic cardiomyopathy (HCM) was admitted for increasing dyspnea and thoracic pain. An acute coronary syndrome was excluded. 2D echocardiography demonstrated LV hypertrophy (Fig. 4, Additional file 2, Additional file 3). She further underwent a CMR (Fig. 5, Additional file 4), demonstrating a systolic narrowing of the LVOT (Fig. 5, Additional file 4). Flow acceleration in the LVOT was detected with CW Doppler (Fig. 5). Of note, the color Doppler (Additional file 2) and classical contrast-enhanced echocardiography (Fig. 4) were unable to identify this feature. One single HFR echoPIV acquisition (Fig. 5, Additional file 5) identified the LVOT flow acceleration and narrowing. The patient improved clinically with medication (beta blocker).

## Patient 2: Mid-LV flow acceleration and apical stasis because of constrictive pericarditis.

**Fig. 6:**
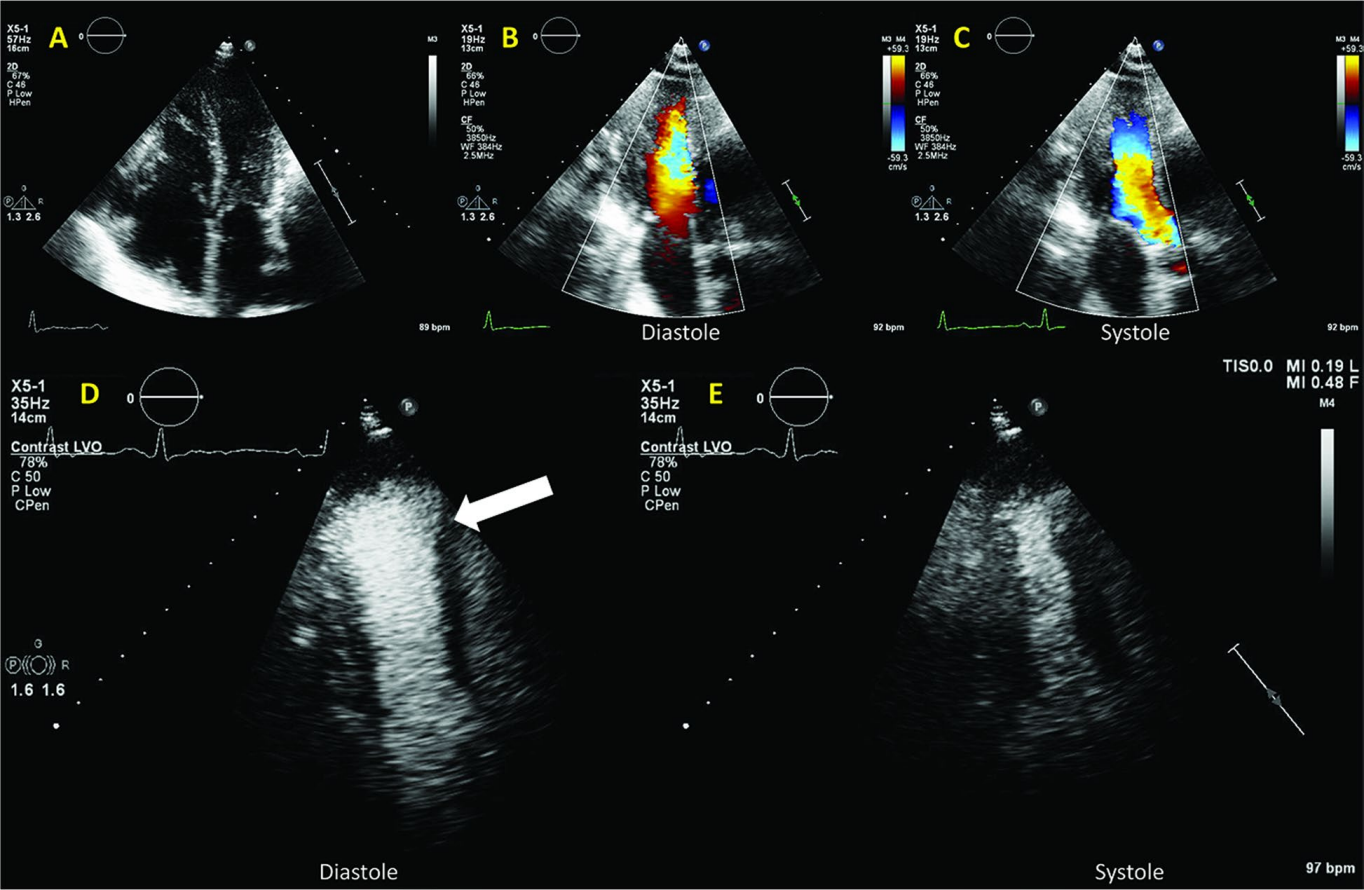
Patient 2. A: 2D echocardiography: LV compressed dilated RV, but with normal systolic function; signs of pressure and volume overload; B, C: Color Doppler of the LV in diastole (B) and systole (C). D, E: clinical contrast in diastole (D) and systole (E). The LV in diastole (arrow) forms a diastolic dilatation of a rather hyperkinetic apex; there is also a near-collapse of the mid-LV cavity

A 65-year-old male patient, operated 9 years before for idiopathic constrictive pericarditis (partial pericardial resection), with known right ventricular (RV) dysfunction and pulmonary hypertension, was admitted for right heart decompensation. Transthoracic echocardiography demonstrated a dilated and dysfunctional RV (Fig. 6).

**Fig. 7:**
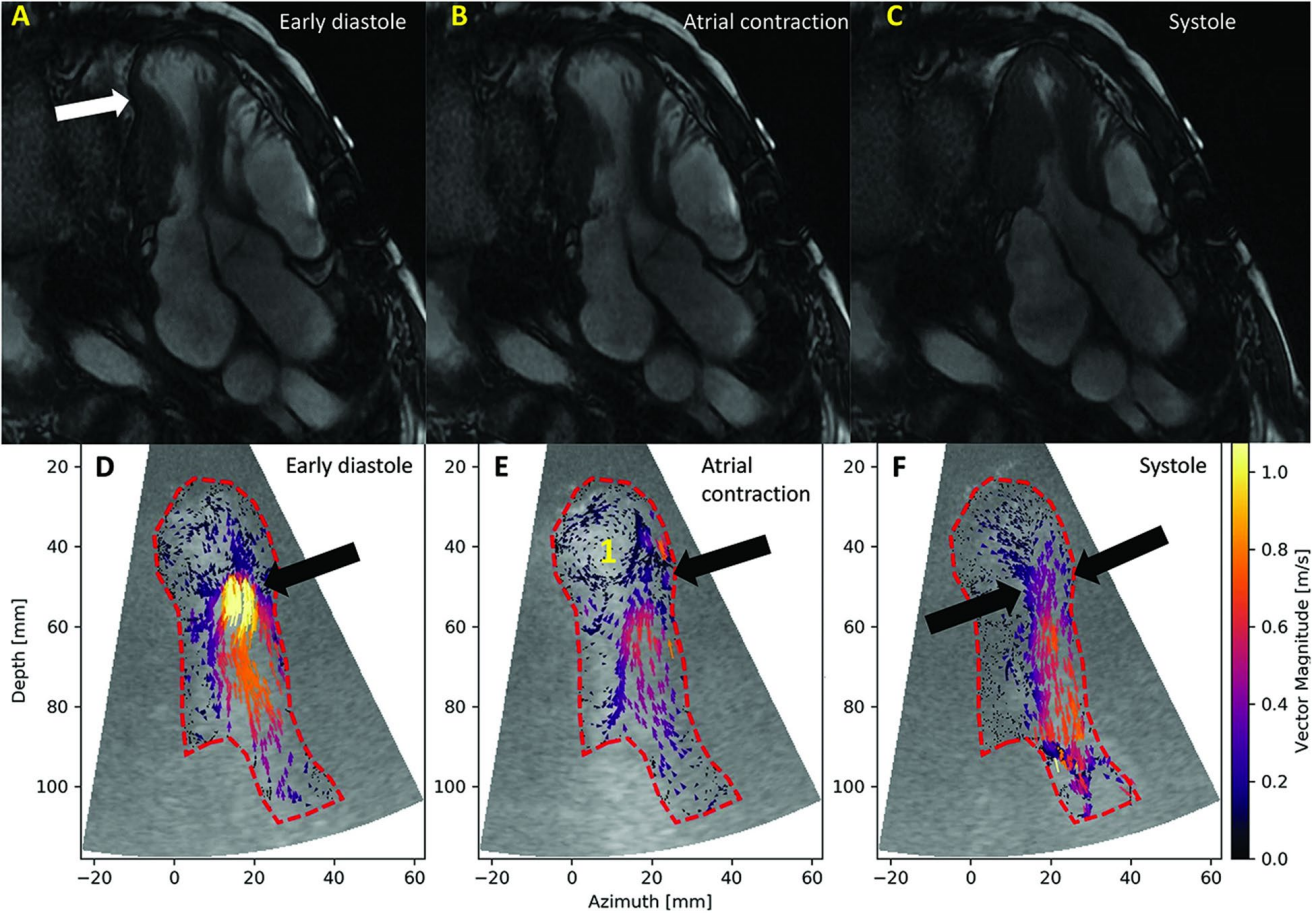
Patient 2. A, B, C: CMR mid-LV compression and apical dilation (arrow); D, E: HFR echoPIV flow acceleration in diastole (black arrow), apical swirling and counter-clockwise vortex (1); F: systolic mid-LV flow acceleration (black arrows)

Classical LV contrast demonstrated a peculiar “mushroom” shape of the LV (Fig. 6; Additional file 6). The patient underwent a CMR (Fig. 7), which explained the LV shape (Additional file 7) by the remnants of rigid calcified pericardium up to the mid third and by septal shift. HFR echoPIV added functional data that was inaccessible through color Doppler: flow acceleration in the mid-LV narrowing, as well as apical swirling and counter-clockwise vortex (Fig. 7, Additional file 8) suggestive of apical low flow during diastole. Given the age and advancement of the disease, the local heart team decided for conservative management. Patient improved clinically with diuretics; he was already on oral anticoagulants.

## Patient 3: Intracavitary energy loss in dilated cardiomyopathy with multiple low-velocity vortices.

**Fig. 8:**
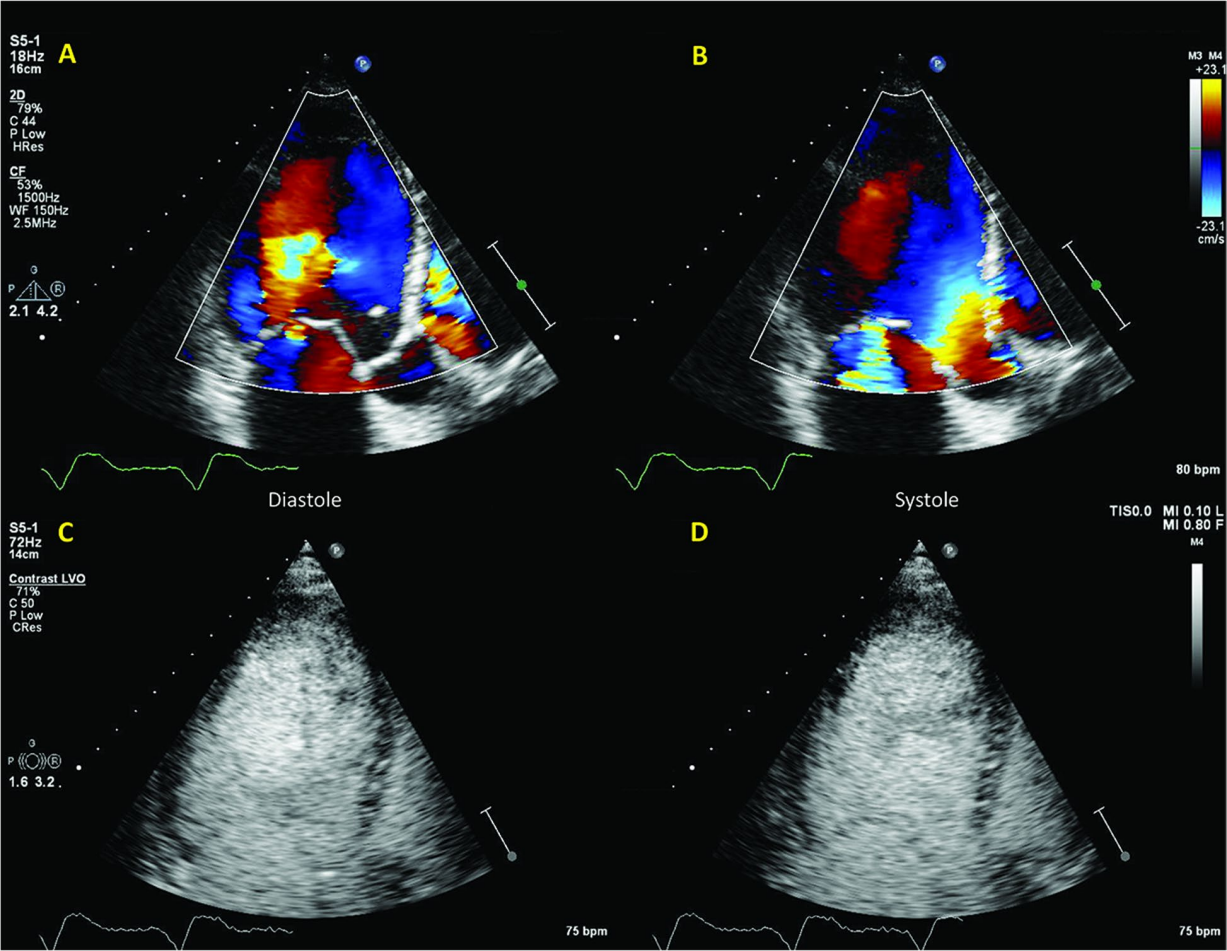
Patient 3. A, B: Color Doppler LV suggested clockwise rotating flow with low velocity (Additional file 9). Note the low color velocity scale, indicating low flow; C, D: Clinical LV contrast imaging demonstrated the same rotational flow, with apical swirling (Additional file 10)

A 62-year-old male patient with known ischemic dilated cardiomyopathy, was admitted for rapid progression of heart failure symptoms, important fluid retention, class III NYHA dyspnea and worsening kidney function. The patient was already on optimal medication including anticoagulants, CRT, physical training and close follow-up. Echocardiography demonstrated a dilated LV, with EF = 22%, restrictive mitral insufficiency and low output (Fig. 8, Additional file 9).

**Fig. 9:**
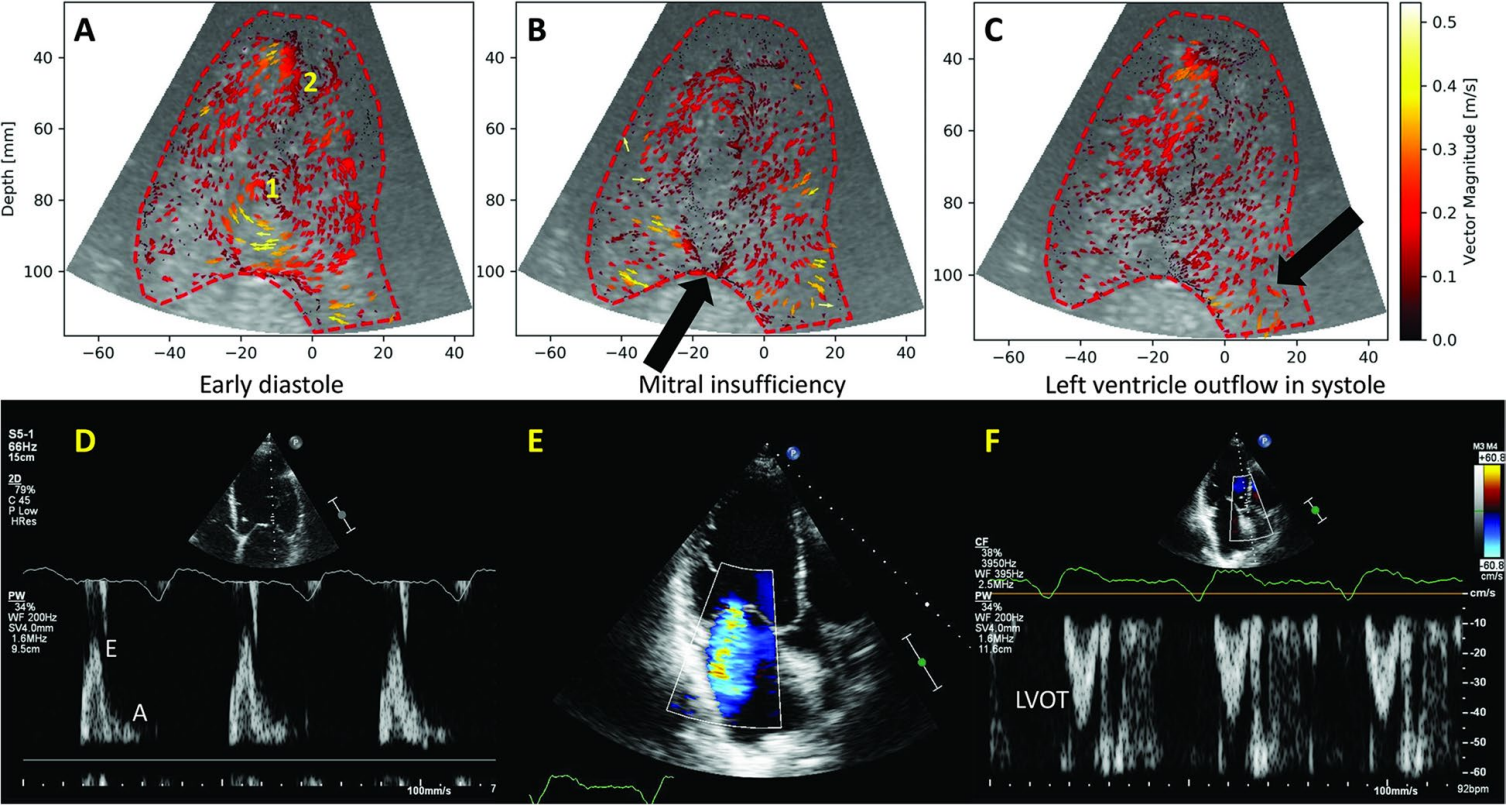
Patient 3. A: There were two main diastolic vortices (1,2), triggered by the restrictive mitral inflow and intraventricular recirculation of flow. Both vortices rotated clockwise, generating a conflicting flow at the interface between them, with mid-LV stasis and increased recirculation in the apex (Additional file 11). (D); B: In systole, a part of the downwards-going flow converged into the mitral insufficiency jet, while the outflow tract displayed low-velocity flow (black arrow; E color Doppler); C: low-velocity LVOT flow (black arrow); F: LVOT PW Doppler confirms the low flow

HFR echoPIV (Fig. 9) noted phenomena that could not be detected with color Doppler or classical contrast echocardiography (Additional file 10). Two main diastolic clockwise rotating vortices were seen (Additional file 11). Part of the systolic flow converged into the mitral insufficiency. This revealed a vicious circle of energy-inefficient contraction and intraventricular conflicting flow. The patient received intravenous diuretics and Levosimendan, with improvement in kidney function. He was enlisted for LVAD/heart transplantation screening.

## Patient 4: Mitral inflow limited by the aortic insufficiency jet.

**Fig. 10:**
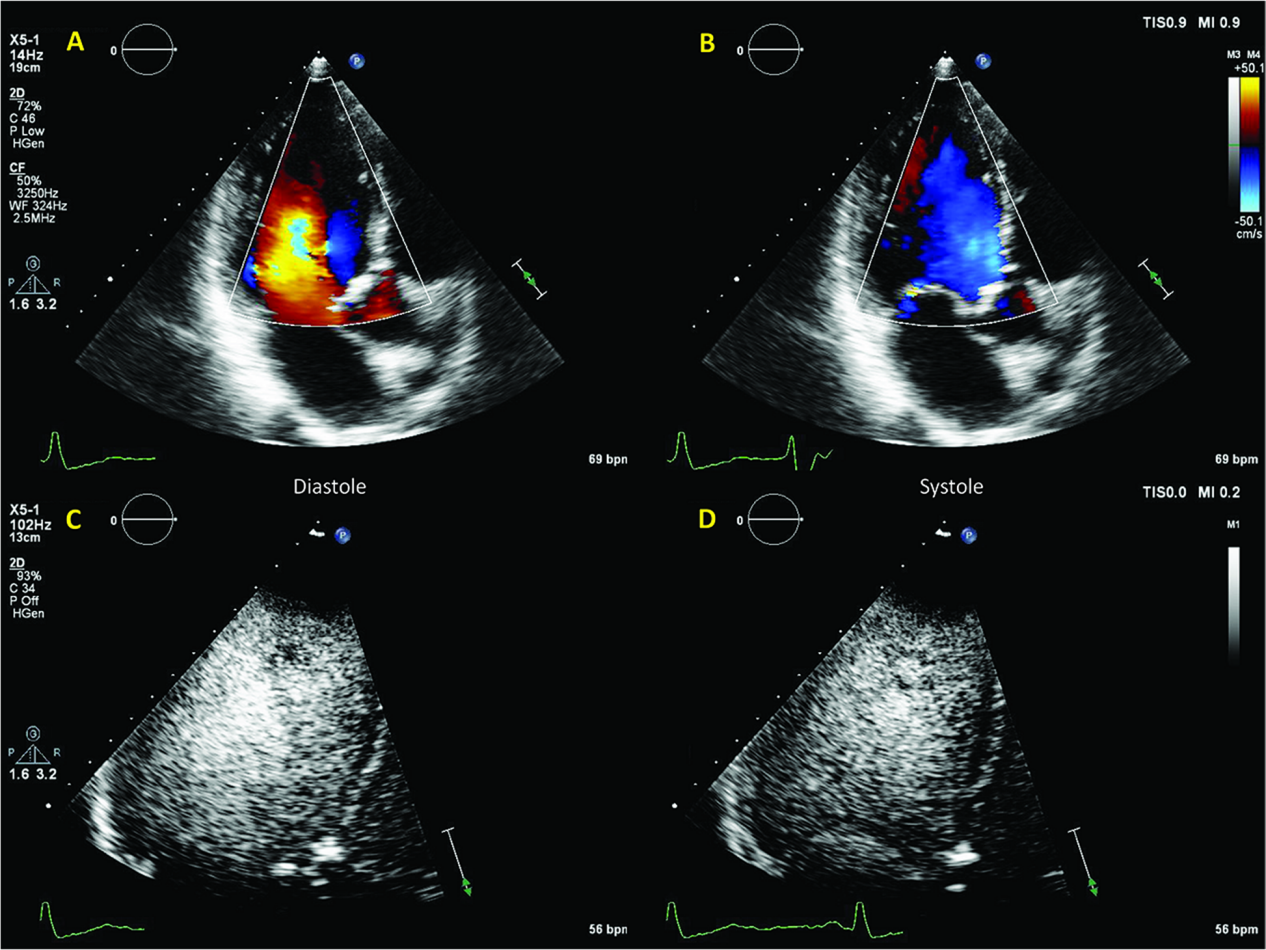
Patient 4. A, B: color Doppler of the LV. Dilated LV and low-flow. The AI jet is not clear; C, D: clinical contrast imaging not diagnostic in spite of good image quality

A 69-year-old male patient was admitted for dyspnea and fatigue. In the last year he had an important weight loss (15 kg), with persistent fatigue and unexplained diarrhea. He had recently been diagnosed with hypereosinophilic syndrome in the referring hospital. On echocardiography LV was dilated with EF = 29%, with calcified moderate aortic stenosis and insufficiency (AI). The AI seemed only trivial at first analysis (Fig. 10; Additional file 12).

**Fig. 11:**
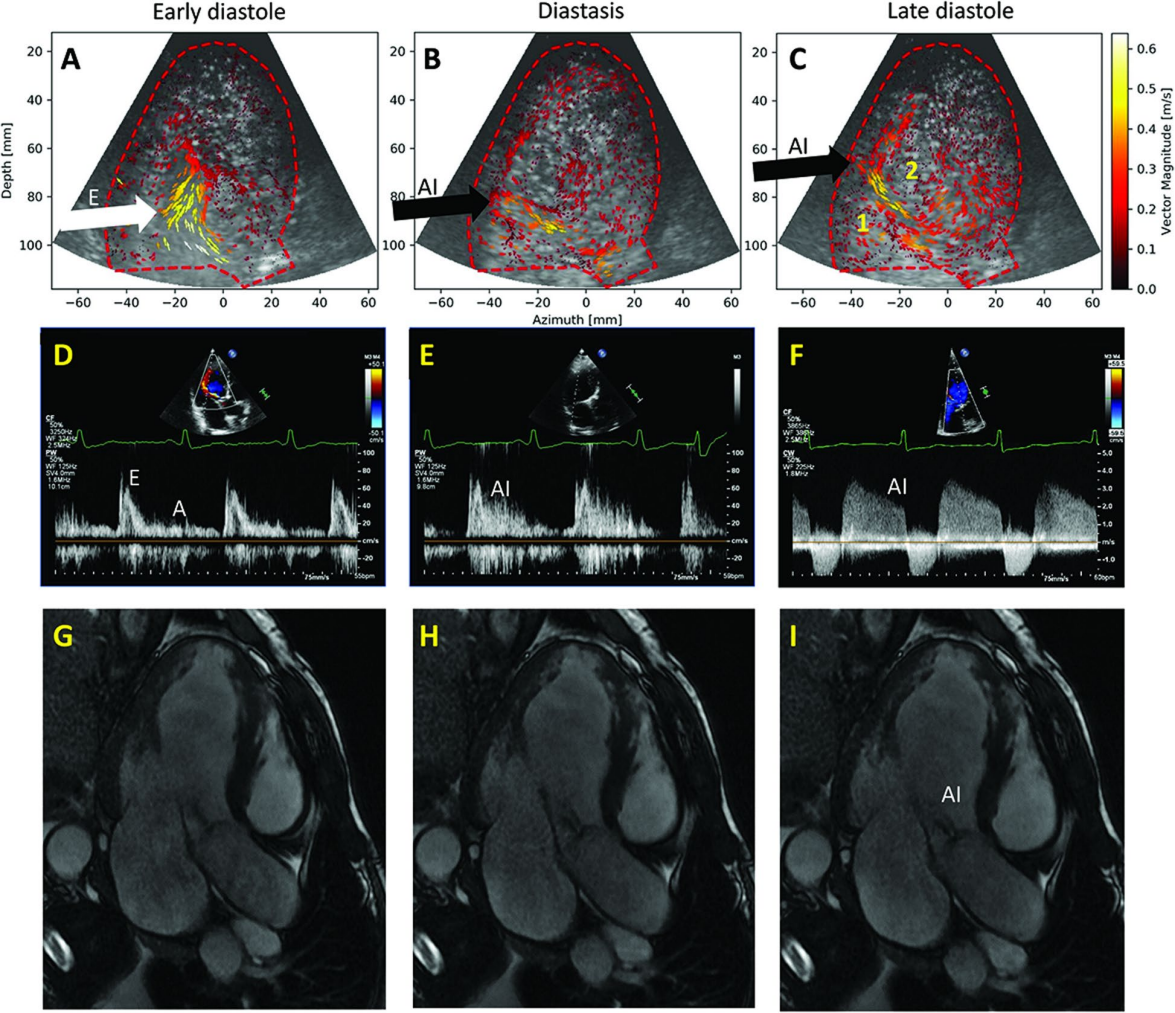
Patient 4. A-C: HFR echoPIV shows an AI jet having a perpendicular course to the ultrasound beam, making it harder to assess with colour Doppler. A local counter-clockwise vortex formed under the mitral valve (C:1) and another clockwise vortex formed in the mid-LV (C:2), leading to recirculation of blood from the AI and mitral inflow high-velocity AI jet limiting the mitral inflow (white arrow); D-E: PW mitral inflow, interference with the AI; F: AI jet on CW Doppler; G-I: CMR, confirming the AI jet pushing the anterior mitral valve

Clinical contrast images were non-contributive (Additional file 13). The patient underwent a CMR (Fig. 11) for the possible eosinophilic myocarditis.

On HFR echoPIV a high-velocity AI jet was seen interfering with filling (Fig. 11, Additional file 14). A counter-clockwise vortex formed under the mitral valve and a clockwise one in the mid-LV (Additional file 14). The AI directed towards the mitral valve was confirmed on CMR (Additional file 15). However, CMR was not highly suggestive of eosinophilic myocarditis. Therefore, a heart biopsy was performed, confirming eosinophilic infiltrate. The patient was started on Prednisone, with clear improvement in symptoms and LV function (one year later EF = 38%, and valve disease remained moderate for both lesions).

## Discussion

We demonstrate the clinical application of high frame rate echoPIV in a wide range of pathologies, in patients with clinical heart failure. The flow velocity estimation through echoPIV was highly correlated with the values derived from regular clinical PW Doppler. However, echoPIV tended to underestimate peak velocities, coming closer to the mean velocities as determined by PW Doppler. In selected patients, echoPIV was able in a single acquisition to demonstrate flow patterns which required multiple interrogations with classical echocardiography. Those flow patterns could also be linked to anatomical abnormalities as seen in CMR or CT.

In this heterogeneous patient population, the tracking quality through echoPIV was similar to the 2D image quality, meaning that as long as 2D images can be obtained, flow tracking can also be performed. Naturally, as for classical contrast imaging, HFR echoPIV is bound to the same set of limitations as echocardiography in general [10]. The study of the inflow region in the apical long axis view was more feasible than the outflow. This may be explained by the position of the LV outflow tract deeper in the image, leading to increased attenuation and clutter.

When quantitatively compared with PW Doppler, we noted that echoPIV underestimated peak velocities, the bias increasing with the velocity magnitude, as demonstrated by the regression slopes in Fig. 3. This trend was also observed by using high frame rate blood speckle-tracking [11]. This underestimation is logical, because the two methods estimate a bulk displacement in the interrogation kernel, and use averaging across a number of frames (Fig. 1), while Doppler displays the maximal velocity in the same region. We therefore compared echoPIV with the mean PW velocity tracing, which resulted in closer correlations and smaller bias, both more evident for the LV inflow.

In general, the total information that could be gathered by using a single-acquisition HFR echoPIV was superior to both conventional echocardiography with Doppler and conventional contrast-enhanced echocardiography, as illustrated by the case examples.

It is also noteworthy that in selected patients abnormal intracavitary jets were seen, that otherwise needed interrogation from multiple conventional echocardiography views and careful Doppler placement (Fig. 4 to Fig. 11), the findings being confirmed with reference imaging tests. These clinical examples show that HFR echoPIV has the ability to pick up high velocity events. Its immediate clinical significance resides mainly in that it can provide in one acquisition all the functional information detected by routine echocardiography with a multiplane approach, color, PW and CW Doppler and classical contrast-enhancement, complemented by multi-modality imaging.

The intricate HFR echoPIV flow fields may offer additional meaningful insights, such as derived quantities (vorticity, circulation, kinetic energy, kinetic energy dissipation, and relative pressure gradients) [1, 2, 11–16]. The precise clinical meaning of these derived parameters still needs to be investigated [14]. More detailed analysis in larger studies need to be performed to ascertain which clinically useful indices can be obtained with HFR echoPIV.

### Limitations and future directions

This feasibility study included only a limited number of patients, with various pathologies. This prevents from obtaining conclusions pertaining to the clinical use of HFR echoPIV in particular diseases. We however benefited from this diversity, which simulated the normal variability encountered in clinical practice.

Although most of the patients had multimodality imaging studies as clinically indicated, this prospective protocol did not include a gold standard method for flow tracking, we only used regular clinical echocardiography, CMR and cardiac CT. We quantitatively compared PW Doppler in only two precise sites in the LV, which had to be situated in line with the Doppler interrogation line (inflow and outflow of the LV).

The present implementation of the HFR echoPIV is limited to 2D HFR echocardiography. The representation of the real intraventricular flow is limited to the position of the slice plane, and cannot detect out-of-plane motion. However, most of the echoPIV studies until now have been performed in 2D, by using the apical long axis view, which intersects the main directions of intraventricular inflow and outflow [14, 16]. We are currently working on an implementation of 3D HFR cardiac imaging, potentially leading to a 3D echoPIV application [17]. An interesting step would be to perform comparative studies with 4D flow CMR, allowing for quantitative and qualitative studies over the whole imaging volume.

As presented in Methods, the boundary mask for the blood flow analysis was fixed, therefore allowing some myocardial signal into the detection region during systole. We are currently working on a semi-automatic endocardial tracking method, making the boundary detection dynamic and adapted to the real LV endocardial contour.

The current version of the software (not optimized for speed) allows for a total duration of this offline postprocessing of approximately 10 min per each recording of 2.5 s. This implies that a clinical application may be developed, in the first step for offline postprocessing, and later for real-time analysis (through parallelization and usage of graphical processing units).

## Conclusion

We demonstrate here the clinical application of HFR echoPIV which succeeds in surpassing the shortcomings of Doppler imaging (angle dependency) and classical contrast echo (low frame rate), with clinical promise and good feasibility in flow tracking and quantification.

## Supplementary Information

**Additional file 1.** HFR echoPIV in a normal subject, in apical longitudinal view. The region of interest is traced along the end-diastolic contours of the LV and include the LVOT. Note the normal diastolic mid-ventricular clockwise vortex induced by the mitral inflow. 

**Additional file 2.** Color Doppler of the LV cavity including the LVOT. In this view the LVOT flow acceleration and narrowing are not visible neither by 2D nor by Color Doppler. 

**Additional file 3. **Classical LV contrast-enhanced echocardiography in the apical long axis view, demonstrating LV hypertrophy and possible narrowing of the LVOT, but the attenuation in the far field precludes optimal visualization.

**Additional file 4.** CMR long axis cine-sequence. LV hypertrophy, systolic narrowing of the LVOT and flow acceleration.

**Additional file 5.** HFR ecoPIV. The systolic outflow (downwards flow through LVOT) presents a narrowing, as well as local acceleration. 

**Additional file 6. **Classical LV contrast-enhanced echocardiography in the apical long axis view, demonstrating mid-LV cavity collapse and apical diastolic dilatation (“mushroom-shaped LV”). The apex has a hyperkinetic motion.

**Additional file 7. **CMR long-axis cine-sequence. The “mushroom” shape is explained by the rigid inferolateral pericardium up to the apical third of the LV (post partial pericardial resection) and by the paradoxical septal motion induced by RV overload. The apex is hyperkinetic and dilated in diastole.

**Additional file 8.** HFR echoPIV. There is systolic and diastolic flow acceleration at the junction between the mid and apical LV. Despite the hyperkinetic apex, there is swirling and low velocity counter-clockwise vortex in the dilated apical LV.

**Additional file 9.** Color Doppler of the LV cavity in apical long axis view. The intracavitary flow seems to have a general clockwise rotation towards LVOT, with low velocity (note the velocity scale on the right). MI is present.

**Additional file 10.** Classical LV contrast-enhanced echocardiography in the apical long axis view. The same global rotational flow is seen as in Color Doppler, with swirling and low flow. MI is not detectable in the far field. 

**Additional file 11.** HFR echoPIV. Two conflicting diastolic vortices rotate clockwise. There is global low flow, both in diastole and in systole. In systole, a part of the downward-going flow converges into the mitral insufficiency. 

**Additional file 12.** Color Doppler of the LV cavity in apical long axis view. Dilated LV with altered systolic function. A mild mitral insufficiency is observed, as well as a seemingly very mild aortic insufficiency. 

**Additional file 13.** Classical LV contrast-enhanced echocardiography in the apical long axis view. Despite a good visualization of the LV structures, no extra information on flow can be deduced.

**Additional file 14.** HFR echoPIV. The mitral inflow is impinged by a high-velocity aortic insufficiency, leading to the formation of multiple vortices and recirculation of the intraventricular flow. Systolic flow velocity in the LVOT is relatively low, accelerating towards the calcified aortic valve.

**Additional file 15. **CMR long-axis cine-sequence. The presence, magnitude and direction of the AI as observed on the echoPIV are confirmed.

## Data Availability

The datasets generated and/or analysed during the current study are not publicly available due to patient privacy but are available from the corresponding author on reasonable request.

## Abbreviations

ALAX: Apical long axis
Cardiac CT: Cardiac computed tomography
CMR: Cardiac magnetic resonance
echoPIV: Echo-Particle Image Velcimetry
HFR: High frame rate
LV: Left ventricle
PW: Pulsed-wave
SD: Standard deviation
UCA: Ultrasound contrast agent
